# Advantages and pitfalls of using free-hand sections of frozen needles for three-dimensional analysis of mesophyll by stereology and confocal microscopy

**DOI:** 10.1111/j.1365-2818.2008.02079.x

**Published:** 2008-10

**Authors:** Z LHOTÁKOVÁ, J ALBRECHTOVÁ, J JANÁČEK, L KUBÍNOVÁ

**Affiliations:** * Charles University in Prague, Faculty of Science, Department of Plant PhysiologyViničná 5, CZ-12844, Prague 2, Czech Republic; † Department of Biomathematics, Institute of Physiology, v.v.i., Academy of Sciences of the Czech RepublicVídeňská 1083, CZ-14220, Prague 4, Czech Republic; ‡ Institute of Botany, v.v.i., Academy of Sciences of the Czech RepublicCZ-25246 Průhonice, Czech Republic

**Keywords:** Confocal microscopy, fakir method, internal surface area, mesophyll, Norway spruce, stereology

## Abstract

The anatomical structure of mesophyll tissue in the leaf is tightly connected with many physiological processes in plants. One of the most important mesophyll parameters related to photosynthesis is the internal leaf surface area, i.e. the surface area of mesophyll cell walls exposed to intercellular spaces. An efficient design-based stereological method can be applied for estimation of this parameter, using software-randomized virtual fakir test probes in stacks of optical sections acquired by a confocal microscope within thick physical free-hand sections (i.e. acquired using a hand microtome), as we have shown in the case of fresh Norway spruce needles recently. However, for wider practical use in plant ecophysiology, a suitable form of sample storage and other possible technical constraints of this methodology need to be checked. We tested the effect of freezing conifer needles on their anatomical structure as well as the effect of possible deformations due to the cutting of unembedded material by a hand microtome, which can result in distortions of cutting surfaces. In the present study we found a higher proportion of intercellular spaces in mesophyll in regions near to the surface of a physical section, which means that the measurements should be restricted only to the middle region of the optical section series. On the other hand, the proportion of intercellular spaces in mesophyll as well as the internal needle surface density in mesophyll did not show significant difference between fresh and frozen needles; therefore, we conclude that freezing represents a suitable form of storage of sampled material for proposed stereological evaluation.

## Introduction

The internal leaf structure is tightly connected with many important physiological processes in leaves (Nobel, 1976; Chabot & Chabot, 1977). Recent studies show that relationships between leaf anatomy parameters and photosynthesis are important in leaf acclimation to high or low irradiances (Robakowski *et al*., 2004; Pandey & Kushwaha, 2005) or elevated CO_2_ concentrations (Eguchi *et al*., 2004).

Conifer needle anatomy has been predominantly studied for Norway spruce (*Picea abies* L. Karst.) since the 1980s mainly due to a widespread forest decline caused by atmospheric pollution and acid rain (e.g. Albrechtová*et al.*, 2001). Norway spruce is widely planted coniferous species in Central and Northern Europe and it is worth developing effective methods for assessment of such needle geometrical characteristics, which may be useful for interpretation of physiological measurements or three-dimensional (3D) modelling (Ustin *et al*., 2001; Aalto & Juurola, 2002; Juurola *et al*., 2005).

The first quantitative descriptions of leaf anatomy (for review see Pazourek, 1988) were based on counting planar features, especially counting stomata per unit area of the leaf (stomata density) or linear measurements, such as the leaf thickness or stoma length assessment. In the first half of the 20th century, model-based methods for quantification of internal leaf structure, like cell volume and cell surface area per unit volume of leaf tissue, emerged (Turrell, 1936). Later on, the development of stereological methods brought new approaches that could be applied to quantitative analysis of internal leaf structure (Weibel, 1979). The point-counting method for measuring volume density or volumetric proportion of leaf tissues has become widely used (Parkhurst, 1982; Albrechtová & Kubínová, 1991; Kubínová, 1991, 1993). For surface area measurements, such as cell surface area exposed to intercellular spaces (i.e. internal leaf surface area, usually expressed per unit leaf area), corresponding stereological methods were established; however, some assumptions about the cell shape were often made, e.g. a specific shape factor for the given population of cells had to be applied (Lee *et al*., 2000; Slaton & Smith, 2002).

Design-based, assumption-free stereological methods, enabling unbiased evaluation of the structure of 3D objects of arbitrary shapes, however, were developed and adapted to evaluate leaf tissues. Our proposal to estimate internal leaf surface area using a design-based stereological method of vertical sections (Kubínová, 1991, 1993) was not used by other authors, probably due to its laboriousness. The more recent methods, based on generation of virtual test probes applied to 3D image data (Kubínová & Janáček, 1998; Larsen *et al*., 1998), acquired, e.g. by a confocal microscope, could be accepted by plant biologists more widely due to a number of advantages: They are efficient, unbiased and can be applied to thick fresh tissue sections, thus minimizing time spent by preparation of tissue specimens.

As we have shown recently (Albrechtová*et al*., 2007), the stereological methods based on virtual test probes applied to 3D image data captured by a confocal microscope can be used for evaluation of mesophyll structure of conifer needles, namely the mesophyll cell number in a needle can be estimated by the optical disector method (Gundersen, 1986) and internal needle surface area (defined as the surface area of mesophyll cell walls exposed to intercellular spaces) by the fakir method (Kubínová & Janáček, 1998). Unlike classical stereological methods applied to thin physical sections, this method for surface area estimation does not require randomizing the orientation of the section; hence, the physical thick sections can be cut in arbitrary direction. Therefore, the slices can be cut perpendicular to the main axis of the needle, which is the most suitable direction from the technical point of view.

The majority of studies on foliar internal structure use sections of fixed and embedded plant material (e.g. Turrell, 1936; Eguchi *et al*., 2004; Pandey & Kushwaha, 2005) but during fixation or embedding the tissue deformation and other artefacts may occur (Uwins *et al*., 1993; Dorph-Petersen *et al.*, 2001). Therefore, using free-hand sections (i.e. acquired by a hand microtome) of fresh needles as we have proposed (Albrechtová*et al*., 2007) eliminates such deformation problems. However, using fresh material can limit the method application in experimental research as only few fresh needles can be analysed simultaneously in one day and the trees under study should not be located too far from the place of needle analysis. In the present study, we suggest to solve this problem by freezing the needles and storing them for later analysis. Further, we address another technical problem, resulting in biased measurements, caused by the possible distortions of cutting surfaces of transversely cut needle sections. Such distortions are likely to occur as conifer needles exhibit the arrangement of mesophyll cells in transversally oriented interconnected layers (Esau, 1953).

The aim of the present study was to test the effect of freezing conifer needles on their anatomical structure as well as the effect of possible deformations due to the free-hand cutting of unembedded material by a hand microtome. For this purpose, we studied free-hand thick sections of fresh and frozen Norway spruce needles. In order to check the possible effect due to freezing we compared the proportion of intercellular spaces and internal needle surface density in mesophyll between fresh and frozen needles in different positions along the needle longitudinal axis. The possible loss of mesophyll cells due to the cutting process was tested by comparing the proportion of intercellular spaces estimated in optical sections located in different depths within the thick free-hand needle section. Finally, the effect of deformations due to cutting needles by a hand microtome on the estimation of the needle internal surface area was tested by comparing estimates of internal needle surface density in mesophyll of fresh and frozen needles measured in different depths within the confocal stacks located inside the thick free-hand needle section.

## Material and methods

### Needle collection

Needles were collected in early May 2005 before the bud burst from 20-year-old Norway spruce tree in the Botanical garden of Charles University in Prague. Five sunlit shoots were selected in the middle of the tree crown and needles of the third needle age class were collected. The needles were sampled from the middle part of the shoot. The described sampling procedure was applied in order to keep low variability in measured parameters for properly testing the freezing effect using a low number of samples, instead of e.g. uniform random sampling of needles. Five needles were processed immediately within 1 h after collection (fresh needles), the other five needles were collected from the same shoot and identical position within the shoot and stored at −70°C for 30 days at least before processing (frozen needles).

### Sample preparation

**Fig. 1:**
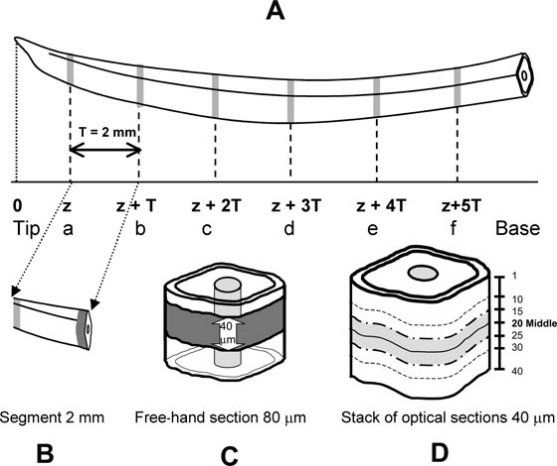
Sampling design of needle specimen preparation. (A) Systematic uniform random sampling of transverse free-hand sections: *z*= random position of the first section within (0; *T*], where *T*= 2 mm. Positions of transverse sections along the needle longitudinal axis are denoted by a, b, c, d, e, f. (B) 2-mm-thick needle segment. thickness was cut. (C) 80-μm-thick free-hand section from which the 40 μm thick stack of optical sections was acquired. (D) Stack of 40 optical sections 1 μm apart, lines refer to optical sections 5 μm apart, where proportion of intercellular spaces in mesophyll was measured.

Transverse free-hand needle sections were cut by a hand microtome (MIC 500, EUROMEX microscopen BV, The Netherlands), which consisted of a piston screwed into a tube whose free end was fixed into a flat plate serving as a stage. The knurled head at the bottom of the instrument moved the piston up or down the central tube when rotated. The specimen was fixed on the flat top of the piston and sections were cut by drawing a razor over the upper flat surface of the instrument. Sections were cut according to the principle of systematic uniform random sampling (Fig. 1A; Gundersen & Jensen, 1987; Kubínová 1991, 1993). Frozen needles were defrosted naturally immediately before their cutting. The free-hand thick sections from one needle were kept in cooled polystyrene box in distilled water until processed. On average, six sections from each needle about 80-μm thick were cut perpendicular to the longitudinal needle axis at an interval of 2 mm (Fig. 1).

### Image acquisition by confocal microscopy

**Fig. 2:**
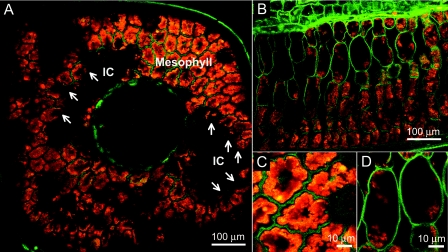
Optical sections of Norway spruce needle acquired by confocal microscopy. Autofluorescence of phenolic acids bond in the cell walls in green, autofluorescence of chlorophyll in red. (A) Transverse section of Norway spruce needle. Mesophyll tissue highlighted, IC = intercellular spaces, white arrows show the cell surface exposed to intercellular spaces which was measured as internal needle surface. (B) Longitudinal needle section. (C) Detail of the mesophyll cell in the transverse section. Note the irregularity of the cell shape. (D) Detail of the mesophyll cell in the longitudinal section.

Images of optical sections of Norway spruce needles were acquired by a Leica SP2 AOBS confocal laser scanning microscope (Leica Microsystems, Wetzlar, Germany) using a 20× water immersion objective (planapochromat, N.A. = 0.7). Images were captured under Ar laser excitation at the wavelength of 488 nm and emission wavelengths at 500–535 nm (first channel) and 598–706 nm (second channel), see Fig. 2. No additional fixation or staining techniques were used – only autofluorescence of chlorophylls and phenolic compounds in the cell walls was exploited.

From the free-hand transverse sections of 80-μm thickness, series of 40 optical sections, 1 μm apart, were captured (Fig. 1C and D) in a following manner: From the very top of the section, a 10-μm-thick guard zone was considered. The images were not acquired from this zone because of apparent cut plane distortions. Then the series of 40 optical sections was captured. It was usually possible to focus through a further 10-μm-thick layer into the depth of the specimen; however, these images were too dark for a proper measurement, due to the fluorescence signal attenuation. The remaining 20-μm-thick layer of the physical section was even darker and practically no fluorescence signal from this region could be detected. Only two neighbouring series were sufficient to encompass entire transverse needle section. After composing the two neighbouring series together, square windows with dimensions of 146.5 μm × 146.5 μm were systematically sampled from each needle section using ‘Rectangles’ module running in Ellipse 2.05 software environment (ViDiTo, Slovakia). The interval between their upper left corners was 256.5 μm in both the vertical and horizontal directions. The sampled substacks of optical sections (146.5 μm × 146.5 μm × 40 μm) were used in all measurements. On average, 70 (±12.4) sampling substacks per needle were measured.

### Measurements

In order to check the possible effect due to freezing, we compared the proportion of intercellular spaces and internal needle surface density in mesophyll between five fresh and five frozen needles in the sections located in different positions along the needle longitudinal axis (a, b, c, d, e, f, as shown in Fig. 1A).

The possible loss of mesophyll cell tissue due to the cutting process was checked by measuring the proportion of intercellular spaces in five optical sections located in different depths within the thick free-hand needle section, namely at 10, 15, 20, 25 and 30 μm within a stack of 40 optical sections 1 μm apart (Fig. 1C and D).

The effect of deformations due to cutting needles by a hand microtome on the measurement of needle internal surface area was tested by comparing estimations of internal needle surface density in mesophyll of fresh and frozen needles measured within five confocal stacks located in different depths within the specimen, as shown in Fig. 1D, namely from 1 to 10 μm, 10 to 15 μm, 15 to 25 μm (i.e. middle 10 μm), 25 to 30 μm and from 30 to 40 μm within a stack of 40 optical sections 1 μm apart.

Volume density of intercellular air spaces in mesophyll was measured by a point-counting method (square point grid with 14 μm distance between the points). On average 371 (±71.0) points for intercellular spaces per needle were recorded. The surface area of mesophyll cells facing the intercellular air spaces (i.e. internal needle surface area) was measured using the fakir method (Kubínová & Janáček, 1998), when the number of intersections between the surface of mesophyll cell walls exposed to intercellular spaces and an isotropic spatial grid consisting of a combination of linear probes called fakir probes was counted (the grid constant, i.e. the distance between neighbouring fakir lines was 27 μm). On average, 288 (±74.2) intersections between fakir probe and the mesophyll surface per needle were recorded. The internal needle surface area was related to the volume unit of mesophyll and expressed as internal needle surface density. The parameters of both point counting and fakir test system were kept constant across all samples in the study.

Statistical analysis was performed using paired *t*-test, one-way anova, nested anova, α level = 0.05 and Tukey–Kramer multiple comparison test.

## Results

**Fig. 3:**
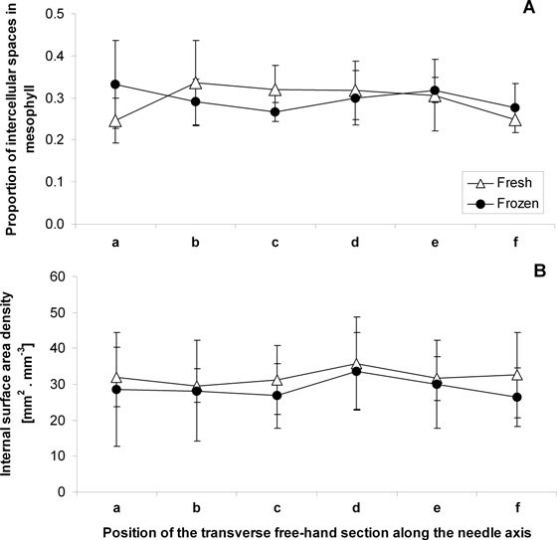
Mesophyll geometrical parameters in different positions along the needle axis from the needle tip (a) to the needle base (f) compared between fresh and frozen needles. (A) Proportion of intercellular spaces in mesophyll, *P*-values and 95% confidence intervals for differences between fresh and frozen needles in individual positions along the needle axis: a: *P*= 0.0467, (0.20; 17.00); b: *P*= 0.5304, (−23.22; 14,02); c: *P*= 0.0509, (−11.13; 0.33); d: *P*= 0.5713, (−9.91; 6.31); e: *P*= 0.7554, (−8.79; 11.19) and f: *P*= 0.5129, (−8.04; 13.64). (B) Internal surface area density, *P*-values and 95% confidence intervals for differences between fresh and frozen needles in individual positions along the needle axis: a: *P*= 0.6030, (−20.07; 13.36); b: *P*= 0.8604, (−19.81; 17,31); c: *P*= 0.5430, (−23.92; 14.69); d: *P*= 0.7858, (−20.09; 16.29); e: *P*= 0.7736, (−17.88; 14.31) and f: *P*= 0.3431, (−22.1604; 9.79). Bars refer to standard deviations. *n*= 5 needles were measured within each treatment, one-way anova did not show significant difference between fresh and frozen needles.

Mean proportion of intercellular spaces in mesophyll was 0.30 (±0.037) for fresh and 0.30 (±0.020) for frozen needles. Mean internal needle surface density in mesophyll was 35.2 (±6.34) mm^2^. mm^−3^ for fresh and 30.2 (±8.76) mm^2^. mm^−3^ for frozen needles. Both selected geometrical parameters did not significantly differ between fresh and frozen needle samples (proportion of intercellular spaces: *P*-value = 0.9631, 95% confidence interval for difference (−6.88; 6.64) and internal surface density: *P*-value = 0.3900, 95% confidence interval for difference (−19.46; 9.36), which suggests that freezing does not alter the needle mesophyll tissue structure and could be used for sample storage. Further, we found almost no effect of freezing on both parameters if tested separately for different positions along the needle longitudinal axis (Fig. 3). Moreover, according to one-way anova proportion of intercellular spaces (*P*-value = 0.2152 for fresh, *P*-value = 0.5458 for frozen needles) and internal needle surface density in mesophyll (*P*-value = 0.9405 for fresh, *P*-value = 0.9404 for frozen needles) did not change along the needle longitudinal axis in both fresh and frozen samples.

**Fig. 4:**
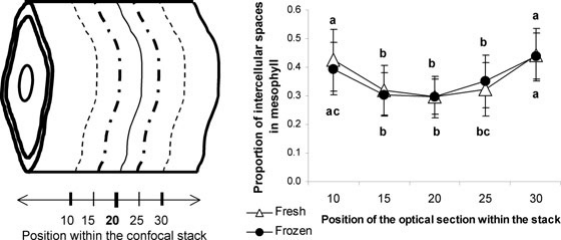
Proportion of intercellular spaces in mesophyll measured in optical sections at different depths of the confocal stack. Numbers on *x*-axis correspond to the order of the optical section within the 40-μm-thick stack of optical sections. Bars refer to standard deviations, different letters show significant differences based on one-way anova. There is no significant difference between fresh and frozen needles, based on paired *t*-test, α= 0.05.

The possible deformation due to the hand cutting was tested and statistical analysis revealed that the proportion of intercellular spaces was significantly higher (*P*-value = 0.0000 for both fresh and frozen) in depths near to the edges of a confocal stack (positions 10 and 30) than in the middle (positions 15, 20 and 25; see Fig. 1). The same effect was observed for both fresh and frozen needles (Fig. 4). This finding suggests that measurement of structural mesophyll parameters should be restricted only to a thin layer inside the thick physical transverse section.

**Fig. 5:**
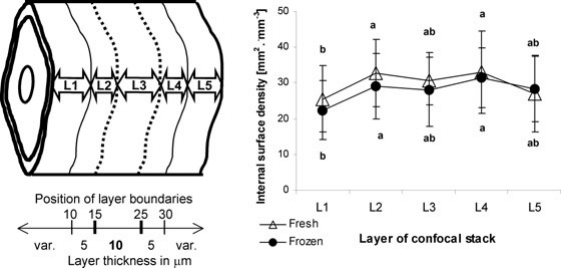
Needle internal surface area density measured in five layers in different depths within the whole 40-μm-thick stack of optical sections. L1–L5 on *x*-axis correspond to individually measured layers. Bars refer to standard deviations, different letters show significant differences based on one-way anova. There is no significant difference between fresh and frozen needles, based on paired *t*-test, α= 0.05.

A similar test was applied in the case of the internal needle surface density in mesophyll of fresh and frozen needles, when the internal needle surface density was measured in five layers (stacks of optical sections) in different depths within the whole series of optical sections (Fig. 5). Although we found differences in internal needle surface density between the layers in different depths within the confocal stack (*P*-value = 0.0099 for fresh, *P*-value = 0.0041 for frozen), the effect of the tissue loss on the cut planes of the physical section was not as obvious as for the proportion of intercellular spaces. However, the internal needle surface density distribution within the confocal stack was identical for fresh and frozen needles.

## Discussion

It is known that during freezing of plant tissues, cellular water migrates to extracellular ice, causing cell dehydration and cell collapse (Levitt, 1980). However, many woody tissues with rigid cell walls have been shown to resist cell collapse during freezing (Malone & Ashworth, 1991), which was our precondition to test freezing as possible storage method for anatomical study of Norway spruce needles. Our studies of technical aspects of using free-hand sections of frozen needles for three-dimensional analysis of mesophyll by stereology and confocal microscopy did not reveal significant differences in all measured characteristics between fresh and frozen needles, i.e. needles stored at −70°C for 30 days at least before processing. This implies that frozen material can be used for extensive ecophysiological studies of needle structure at different locations, even far from the place of needle analysis. Relatively wide confidence intervals for difference in proportion of intercellular spaces and internal surface density suggest that some small changes caused by freezing may have gone undetected. Probably an extension of the study with larger number of needle samples would help to generate tighter confidence intervals. Still, from the plant anatomist's point of view, the possibility of using frozen stored plant material appears to be an efficient method, as time consuming and laborious process of tissue fixation and embedding (which often brings artefacts as shown by Uwins *et al*., 1993) could be avoided in contrast to studies where classical microtechnical methods were used (Turrell, 1936; James *et al.*, 1999; Slaton *et al*., 2001).

On the other hand, our studies confirmed that the distortions of cutting surfaces of transversely cut needle sections present a real technical problem. The higher proportion of intercellular spaces in mesophyll in optical sections located near to the cutting surface of the thick free-hand needle section (i.e. at 10 μm from the beginning of the confocal stack, see Figs 1D and 4) indicates that some of the mesophyll cells are torn out from the cutting surface. The proportion of intercellular spaces was also higher in optical sections deeper in the thick free-hand section (i.e. at 30 μm from the beginning of the confocal stack, see Figs 1D and 4), which could be explained by the high optical density of needle mesophyll cells preventing to focus into deeper layers of the specimen by a confocal microscope. The measurements thus should be restricted only to a relatively thin layer inside the free-hand section, in our case having thickness of 10–20 μm (Fig. 1D). Interestingly, the distribution of internal surface area density within the thick free-hand needle section was not clearly lower in the layers closer to the cutting surface (Fig. 5). This can be explained by the fact that after some of the mesophyll cells were torn out from the cutting surface, in the remaining cells the area of their walls exposed to intercellular spaces (i.e. not neighbouring with other cell walls) increased, leading to the partial compensation of the loss in internal surface area ascribed to missing cells. However, it is not possible to rely on this kind of compensation which is very difficult to predict in individual cases. Therefore, in general, the rule of performing the measurement only in a thin layer within the thick free-hand needle section should be followed even in the case of internal surface area density estimation. The same holds for other stereological measurements, especially counting mesophyll cells by the optical disector principle (Gundersen, 1986; Albrechtová*et al*., 2007).

Clearly, stereological methods based on 3D test probes, such as fakir and disector methods, applied to 3D image data captured by a confocal microscope can be applied to evaluation of anatomical structure of other plant tissues and organs, e.g. leaves, stems or roots, using their thick free-hand sections cut in the most convenient direction.

The problem of distortions of cutting surfaces should have to be addressed here, too, though it can be expected that the probability of tearing the cells out will be different in different tissues, depending on the arrangement of cells and tissue density. For example, in grass leaves, probably not so many mesophyll cells will be torn out from transverse sections, as they are elongated, having their main axis parallel to the leaf axis. In conifer needles, this problem is more pronounced as the mesophyll cells are arranged in transversally oriented interconnected layers (Fig. 2, Esau, 1953). The optimal thickness and position of the layer within the free-hand section suitable for the measurement thus should be checked for each type of material separately.

Another problem with thick free-hand sections can be with their flattening, i.e. shrinking to lower than original thickness. This does not present a serious problem with the tested plant material, as needle tissues (in particular, epidermal and hypodermal cells) have relatively firm cell walls; however, in fine plant materials (fine herbaceous leaves or fine roots), human and animal tissues the thick sections tend to flatten substantially without any artificial support. The phenomenon of tissue shrinkage together with application of stereological techniques is in detail discussed by Dorph-Petersen *et al.* (2001).

On the one hand, it is important to keep in mind that the precision of the hand microtome is limited – therefore the free-hand sections vary slightly in their thickness; however, in all cases it was possible to acquire confocal stack at least 40-μm thick. On the other hand, increasing thickness of the physical section would not improve confocal images acquisition because of inability of proper focusing deeper into the thick section. This also explains why the series of 40 optical sections 1 μm apart used for stereological measurements was shifted towards one of the cut planes (Fig. 1C). In fact, we were able to detect fluorescence signal through approximately 60 μm of the physical 80-μm-thick free-hand cross-section; however only the selected 40-μm-thick stack was suitable for further application of stereological measurements.

In conclusion, the thick free-hand sections of frozen needles can be suitable for estimation of important mesophyll geometrical characteristics using contemporary stereological methods combined with confocal microscopy acquisition, provided the mentioned technical aspects are taken carefully into account. The clear advantage of such approach is a high efficiency achieved by very fast preparation of specimens for microscopy with tissues not affected by fixation, staining and embedding and other technical processing resulting in tissue shrinkage and other artefacts. Plant leaf material is especially suitable for such a type of analysis using confocal microscopy as autofluorescence of chloroplasts and phenolic compounds localized in plant cell walls and the rigidity of plant tissues composed of cells with firm cell walls can be exploited.

## References

[b1] Aalto T, Juurola E (2002). A three-dimensional model of CO_2_ transport in airspaces and mesophyll cells of a silver birch leaf. Plant Cell Environ.

[b2] Albrechtová J, Kubínová L (1991). Quantitative-analysis of the structure of etiolated barley leaf using stereological methods. J. Exp. Bot.

[b3] Albrechtová J, Rock BN, Soukupová J, Entcheva P, Šolcová B, Polák T (2001). Biochemical, histochemical, structural and reflectance markers of damage in Norway spruce from the Krušné hory used for interpretation of remote sensing data. J. Forest Sci.

[b4] Albrechtová J, Janáček J, Lhotáková Z, Radochová B, Kubnínová L (2007). Novel efficient methods for measuring mesophyll anatomical characteristics from fresh thick sections using stereology and confocal microscopy: application on acid rain treated Norway spruce needles. J. Exp. Bot.

[b5] Chabot BF, Chabot JF (1977). Effects of light and temperature on leaf anatomy and photosynthesis in Fragaria vesca. Oecologia (Berl.).

[b6] Dorph-Petersen K-A, Nyengaard JR, Gundersen HJG (2001). Tissue shrinkage and unbiased stereological estimation of particle number and size. J. Microsc.

[b7] Eguchi N, Fukatsu E, Funada R, Tobita H, Kitao M, Maruyama Y, Koike T (2004). Changes in morphology, anatomy, and photosynthetic capacity of needles of Japanese larch (Larix kaempferi) seedlings grown in high CO_2_ concentrations. Photosynthetica.

[b8] Esau K (1953). Plant Anatomy.

[b9] Gundersen HJG (1986). Stereology of arbitrary particles. A review of unbiased number and size estimators and the presentation of some new ones, in the memory of William R. Thompson. J. Microsc.

[b10] Gundersen HJG, Jensen EB (1987). The efficiency of systematic sampling in stereology and its prediction. J. Microsc.

[b11] James SA, Smith WK, Vogelmann TC (1999). Ontogenetic differences in mesophyll structure and chlorophyll distribution in Eucalyptus Globulus ssp. Globulus (Myrtaceae). Am. J. Bot.

[b12] Juurola E, Aalto T, Thum T, Vesala T, Hari P (2005). Temperature dependence of leaf-level CO_2_ fixation: revising biochemical coefficients through analysis of leaf three-dimensional structure. New Phytol.

[b13] Kubínová L (1991). Stomata and mesophyll characteristics of barley leaf as affected by light – stereological analysis. J. Exp. Bot.

[b14] Kubínová L (1993). Recent stereological methods for the measurement of leaf anatomical characteristics – estimation of volume density, volume and surface area. J. Exp. Bot.

[b15] Kubínová L, Janáček J (1998). Estimating surface area by the isotropic fakir method from thick slices cut in an arbitrary direction. J. Microsc.

[b16] Larsen JO, Gundersen HJG, Nielsen J (1998). Global spatial sampling with isotropic virtual planes: estimators of length density and total length in thick, arbitrarily orientated sections. J. Microsc.

[b17] Lee DW, Oberbauer SF, Johnson P, Krishnapilay B, Mansor M, Mohamad H, Yap SK (2000). Effects of irradiance and spectral quality on leaf structure and function in seedlings of two Southeast Asian Hopea (dipterocarpaceae) species. Am. J. Bot.

[b18] Levitt J (1980). Responses of Plants to Environmental Stresses.

[b19] Malone SR, Ashworth E N (1991). Freezing stress response in woody tissues observed using low-temperature scanning electron microscopy and freeze substitution techniques. Plant Physiol.

[b20] Nobel PS (1976). Photosynthetic rates of sun versus shade leaves for Hyptis emoryi Torr. Plant Physiol.

[b21] Pandey S, Kushwaha R (2005). Leaf anatomy and photosynthetic acclimation in Valeriana jatamansi L. grown under high and low irradiance. Photosynthetica.

[b22] Parkhurst DF (1982). Stereological methods for measuring internal leaf structure variables. Am. J. Bot.

[b23] Pazourek J (1988). The evolution of quantitative plant anatomy. Acta Universitatis Carolinae – Biologica.

[b24] Robakowski P, Wyka T, Samardakiewicz S, Kierzkowski D (2004). Growth, photosynthesis, and needle structure of silver fir (Abies alba Mill.) seedlings under different canopies. Forest Ecol. Manag.

[b25] Slaton MR, Smith WK (2002). Mesophyll architecture and cell exposure to intercellular air space in alpine, desert, and forest species. Int. J. Plant Sci.

[b26] Slaton MR, Hunt RE, Smith WK (2001). Estimating near-infrared leaf reflectance from leaf structural characteristics. Am. J. Bot.

[b27] Turrell FM (1936). The area of the internal exposed surface of dicotyledon leaves. Am. J. Bot.

[b28] Ustin SL, Jacquemoud S, Govaerts Y (2001). Simulation of photon transport in a three-dimensional leaf: Implications for photosynthesis. Plant Cell Environ.

[b29] Uwins PJR, Murray M, Gould RJ (1993). Effects of four different processing techniques on the microstructure of potatoes: comparison with fresh samples in the ESEM. Microsc. Res. Techn.

[b30] Weibel ER (1979). Stereological methods. Practical Methods for Biological Morphometry.

